# Analysis of HIV Diversity in HIV-Infected Black Men Who Have Sex with Men (HPTN 061)

**DOI:** 10.1371/journal.pone.0167629

**Published:** 2016-12-09

**Authors:** Iris Chen, Gordon Chau, Jing Wang, William Clarke, Mark A. Marzinke, Vanessa Cummings, Autumn Breaud, Oliver Laeyendecker, Sheldon D. Fields, Sam Griffith, Hyman M. Scott, Steven Shoptaw, Carlos del Rio, Manya Magnus, Sharon Mannheimer, Hong-Van Tieu, Darrell P. Wheeler, Kenneth H. Mayer, Beryl A. Koblin, Susan H. Eshleman

**Affiliations:** 1 Department of Pathology, Johns Hopkins University School of Medicine, Baltimore, Maryland, United States of America; 2 Vaccine and Infectious Disease Division, Fred Hutchinson Cancer Research Center, Seattle, Washington, United States of America; 3 Laboratory of Immunoregulation, National Institute of Allergy and Infectious Diseases, National Institutes of Health, Baltimore, Maryland, United States of America; 4 Department of Medicine, Johns Hopkins University School of Medicine, Baltimore, Maryland, United States of America; 5 Mervyn M. Dymally School of Nursing, Charles R. Drew University of Medicine and Science, Los Angeles, California, United States of America; 6 Science Facilitation Department, FHI 360, Durham, North Carolina, United States of America; 7 Bridge HIV, San Francisco Department of Public Health, San Francisco, California, United States of America; 8 Department of Family Medicine, University of California Los Angeles, Los Angeles, California, United States of America; 9 Department of Global Health, Emory University Rollins School of Public Health, Atlanta, Georgia, United States of America; 10 Department of Epidemiology and Biostatistics, Milken Institute School of Public Health at The George Washington University, Washington, District of Columbia, United States of America; 11 Department of Medicine, Harlem Hospital, Columbia University, Mailman School of Public Health, New York, New York, United States of America; 12 Laboratory of Infectious Disease Prevention, Lindsley F. Kimball Research Institute, New York Blood Center, New York, New York, United States of America; 13 School of Social Welfare, University at Albany, State University of New York, Albany, New York, United States of America; 14 The Fenway Institute, Fenway Health, Boston, Massachusetts, United States of America; 15 Infectious Disease Division, Beth Israel Deaconess Medical Center, Boston, Massachusetts, United States of America; 16 Department of Medicine, Harvard Medical School, Boston, Massachusetts, United States of America; "INSERM", FRANCE

## Abstract

**Background:**

HIV populations often diversify in response to selective pressures, such as the immune response and antiretroviral drug use. We analyzed HIV diversity in Black men who have sex with men who were enrolled in the HIV Prevention Trials Network 061 study.

**Methods:**

A high resolution melting (HRM) diversity assay was used to measure diversity in six regions of the HIV genome: two in *gag*, one in *pol*, and three in *env*. HIV diversity was analyzed for 146 men who were HIV infected at study enrollment, including three with acute infection and 13 with recent infection (identified using a multi-assay algorithm), and for 21 men who seroconverted during the study. HIV diversification was analyzed in a paired analysis for 62 HIV-infected men using plasma samples from the enrollment and 12-month (end of study) visits.

**Results:**

Men with acute or recent infection at enrollment and seroconverters had lower median HRM scores (lower HIV diversity) than men with non-recent infection in all six regions analyzed. In univariate analyses, younger age, higher CD4 cell count, and HIV drug resistance were associated with lower median HRM scores in multiple regions; ARV drug detection was marginally associated with lower diversity in the *pol* region. In multivariate analysis, acute or recent infection (all six regions) and HIV drug resistance (both *gag* regions) were associated with lower median HRM scores. Diversification in the *pol* region over 12 months was greater for men with acute or recent infection, higher CD4 cell count, and lower HIV viral load at study enrollment.

**Conclusions:**

HIV diversity was significantly associated with duration of HIV infection, and lower *gag* diversity was observed in men who had HIV drug resistance. HIV *pol* diversification was more pronounced in men with acute or recent infection, higher CD4 cell count, and lower HIV viral load.

## Introduction

The high genetic diversity of HIV complicates the use of antiretroviral (ARV) drugs for antiretroviral treatment (ART) and has hindered the development of an HIV vaccine [1,2]. HIV diversity is usually measured by sequencing individual viral variants via cloning [3–6], single genome sequencing [7–10], or next generation sequencing [11,12]. However, the effort and cost of sequence-based analysis may limit study size and the number of genomic regions analyzed. We developed a high resolution melting (HRM) diversity assay that quantifies viral diversity without sequencing; the level of genetic diversity is reported as a single numeric HRM score [13,14]. A previous study provides detailed information about the impact of mutations on HRM scores [15]. In a separate study that included analysis of HIV *env* and *gag* diversity in 220 samples, HRM scores were closely correlated with sequence-based diversity measures (genetic diversity, genetic complexity, and Shannon entropy) obtained by analyzing data from next-generation sequencing [16].

The HRM diversity assay has been used to analyze HIV diversity in adults with recent and established infection and in HIV-infected infants and children [13,17–21]. Because HIV infection is typically initiated by only one or a few HIV variants, HIV diversity is usually low early in infection. HIV diversification begins shortly after infection and is driven by a large viral population, short viral half-life, frequent viral recombination, and error-prone replication [22]. Higher HRM scores (i.e., higher levels of diversity) were observed in adults with a longer duration of HIV infection [17,18] and in older children [19,21], where age is a surrogate for the duration of infection. These findings are consistent with findings from other studies that analyzed HIV diversity using sequence-based measures [9,10].

Selective pressures, such as the host immune response and ARV drug use, also impact HIV diversity. HIV evasion from immune responses may result in higher diversity [23]; however, the relationship between HIV diversity and CD4 cell count remains unclear. An association between higher *gag* diversity and lower CD4 cell count was observed in one study [6], but not others [11,17]. Similarly, some studies have reported an association of higher *gag* or *env* diversity with higher viral load [4,6,7,24], while others have reported no association [13,20,21]. HIV diversity can also be affected by ARV drug use and is associated with clinical outcomes. Lower HRM scores were observed in children who experienced prolonged exposure to non-suppressive ART (i.e., genetic bottlenecking) [19]. In a cohort of African children, higher HRM scores in *pol* were associated with better ART outcomes, including shorter time to virologic suppression and longer time to virologic failure [20]. In contrast, higher HRM scores in *gag* and *pol* were associated with decreased 5-year survival in children who did not receive ART [21].

In this study, we analyzed HIV diversity in Black men who have sex with men (MSM) enrolled in the HIV Prevention Trials Network (HPTN) 061 study [25,26]. HPTN 061 was a cohort study in the United States (US) that assessed the feasibility of a multicomponent intervention to reduce HIV incidence among Black MSM; HIV-infected and HIV-uninfected men were enrolled and followed for one year. The HIV-infected men enrolled in HPTN 061 included men with recent and non-recent HIV infection and men at varying stages of disease progression [25,26]. In addition, retrospective ARV drug testing revealed that many men who reported no past or current ARV drug use were taking ARV drugs for ART or other reasons [27,28], and many of the men had unusual patterns of ARV drugs detected [28]. Many HIV-infected men also had resistance to one or more ARV drugs [28]. We used the HRM diversity assay to evaluate HIV diversity among newly-infected men in the HPTN 061 cohort. This study extends our previous work by examining factors associated with HIV diversity in a cohort with high levels of non-suppressive ARV drug use and HIV drug resistance, and by examining factors associated with HIV diversification.

## Methods

### Study cohort

The HPTN 061 study (NCT 00951249) enrolled 1,553 Black MSM in six US cities: Atlanta, Boston, Los Angeles, New York City, San Francisco, and Washington, DC [25,26]. Men were enrolled between July 2009 and October 2010 and followed for one year. Study recruitment methods and eligibility are described in previous reports [25,26]. Briefly, self-identified Black MSM who reported at least one instance of unprotected anal intercourse in the prior six months were recruited from the community or were referred by their sexual network partners. HIV rapid tests and tests for sexually transmitted infections were performed at the study sites at the enrollment, 6-month, and 12-month study visits. CD4 cell count and HIV viral load were measured for men with HIV infection. Behavioral assessments were administered at each study visit using audio computer-assisted self-interviews. In addition, participants completed demographic and social and sexual network questionnaires with an interviewer.

### Laboratory methods

HIV status was confirmed by retrospective testing at the HPTN Laboratory Center (Johns Hopkins University, Baltimore, MD); this included identification of men with acute infection and confirmation of seroconversion events [25,26]. A multi-assay algorithm (MAA) was used to identify men who were likely to have been infected with HIV in the months prior to enrollment; these men were classified as having recent infection. The MAA used in this study included two serologic assays (the BED-capture enzyme immunoassay and an avidity assay) and two non-serologic biomarkers (CD4 cell count and HIV viral load) [28]. The window period for recent infection using this MAA is 159 days (95% confidence interval [CI]: 134–186 days) [29]. The HRM diversity assay was performed on samples from HIV-infected men who had viral loads >400 copies/mL; this included men who were HIV infected at enrollment and men who seroconverted during the study. Six regions of the HIV genome (ranging in size from 100 to 284 base pairs) were analyzed using the HRM diversity assay: two in *gag* (GAG1 and GAG2), one in *pol* (POL), and three in *env* (ENV1, ENV2, and ENV3) [13,17]; HXB2 coordinates of these six regions and the primers used for HRM analysis are described in a previous report [17]. Briefly, each HRM region was amplified using a nested polymerase chain reaction in the presence of a fluorescent, intercalating, duplex-dependent dye [13]. A LightScanner instrument (Model HR 96, BioFire Diagnostics Inc., Salt Lake City, UT) was used to melt the resulting DNA amplicons. Release of the fluorescent dye was quantified from melting curves produced by the LightScanner software (plotted as -*d*[fluorescence]/*d*[temperature]) [13]. The melting range of the DNA amplicons (i.e., the number of degrees over which melting occurred, HRM score) was calculated from each melting curve using an automated R software package (DivMelt, version 1.02) [14]. ARV drug testing and HIV genotyping were performed in a previous study for HIV-infected men who had viral loads >400 copies/mL [28]. ARV drug testing was performed using a qualitative, high-throughput assay based on high-resolution mass spectrometry; this assay detects 15 ARV drugs: four nucleoside/nucleotide reverse transcriptase inhibitors (NRTIs: emtricitabine, lamivudine, tenofovir, and zidovudine), two non-nucleoside reverse transcriptase inhibitors (NNRTIs: efavirenz and nevirapine), and nine protease inhibitors (PIs: atazanavir, amprenavir, darunavir, indinavir, lopinavir, nelfinavir, saquinavir, tipranavir, and ritonavir) [28,30]. HIV genotyping was performed using the ViroSeq HIV-1 Genotyping System, v2.8 (Celera Diagnostics, Alameda, CA), which predicts HIV drug resistance to NRTIs, NNRTIs, and PIs. Samples were classified as having drug resistance using the ViroSeq Algorithm advisor [28].

### Statistical methods

Median regression analyses were used to evaluate associations between HRM scores and demographic and clinical characteristics for each HRM region; *p*-values <0.05 were considered statistically significant. Wilcoxon signed-rank tests were used to analyze the change in HRM score from enrollment to end of study in a paired analysis. Associations between change in HRM score and demographic and clinical characteristics were also assessed using median regression analyses. Multivariate analysis was performed for men who were HIV infected at enrollment using multiple median regressions; variables that were significantly associated with HRM scores at the *p*<0.05 level in univariate analyses were included. The Benjamini-Hochberg procedure [31] was used to correct for multiple comparisons. The false discovery rate was set at 0.20. All statistical analyses were performed using SAS version 9.4.

### Ethical considerations

Written informed consent was obtained from all study participants in the HPTN 061 study. The institutional review boards at each participating institution approved the study: Fenway Community Health; Harlem Prevention Center; New York Blood Center; George Washington University Medical Center; Emory University; San Francisco Vaccine and Prevention CRS; and University of California Los Angeles, Vine Street.

## Results

### Samples used for analysis

**Fig 1:**
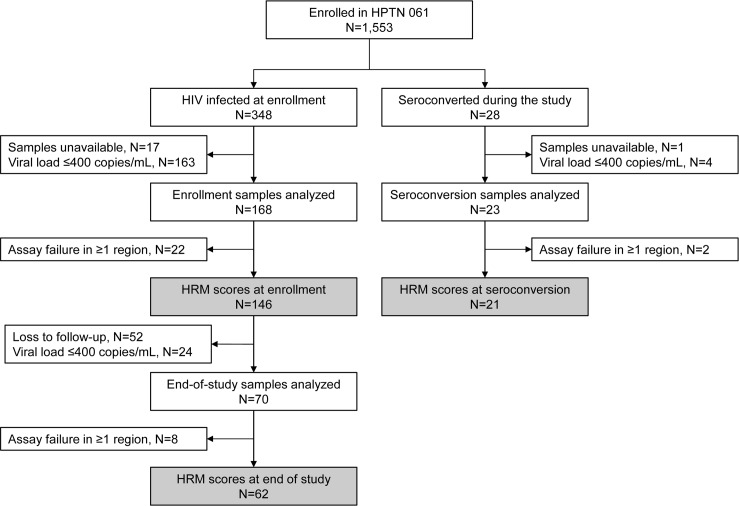
Study cohort. The high resolution melting (HRM) diversity assay was performed for a subset of men in HPTN 061. Data was analyzed for men who had HRM scores determined for all six regions in the HIV genome (shaded boxes).

In HPTN 061, 348 men were HIV infected at study enrollment. The HRM diversity assay was performed for 168 (48%) of these men; the remaining 180 men included 163 men who had a viral load ≤400 copies/mL and 17 men who had no sample available for testing (Fig 1). The assay was also performed for 23 (82%) of 28 men who seroconverted during the HPTN 061 study; the remaining five seroconverters included four men who had viral loads <400 copies/mL at the first HIV-positive visit [30] and one man who had no sample available for testing. HRM scores for all six regions were obtained for 146 (87%) of the 168 men tested who were HIV infected at enrollment and 21 (91%) of 23 seroconverters tested (Fig 1, S1 File). Table 1 shows characteristics of the 167 men included in the analyses.

**Table 1:** Summary of study participants.

Subset	Total	Age (years)^a^	CD4 cell count (cells/mm^3^)^a^	HIV viral load (log_10_)^a^	ARV drug(s) detected^b^	HIV drug resistance^c^
HIV infected at enrollment	146	39 (28, 45)	356 (196, 519)	4.36 (3.75, 4.82)	48 (33%)	42 (29%)
Acute or recent infection	16	25 (20, 36)	525 (381, 804)	4.64 (4.24, 5.29)	10 (63%)	4 (25%)
Non-recent infection	130	41 (31, 46)	321 (193, 499)	4.32 (3.73, 4.81)	38 (29%)	38 (29%)
HIV seroconverters	21	23 (20, 28)	576 (324, 707)	4.90 (4.29, 5.22)	0	5 (24%)

Abbreviations: ARV: antiretroviral.

^a^ The median (interquartile range) for age, CD4 cell count, and HIV viral load are shown.

^b^ The ARV drug assay was used to detect 15 antiretroviral drugs, including nucleoside/nucleotide reverse transcriptase inhibitors (NRTIs), non-nucleoside reverse transcriptase inhibitors (NNRTIs), and nine protease inhibitors (PIs, see Methods). NRTIs, NNRTIs, and PIs were detected in 30, 8, and 29 HIV-infected men at enrollment, respectively.

^c^ Resistance to ARV drugs was assessed using the ViroSeq HIV-1 Genotyping System (see Methods). HIV drug resistance to NRTIs, NNRTIs, and PIs were detected in 20, 35, and 6 HIV-infected men at enrollment, respectively. Four of the seroconverters had HIV resistance to NNRTIs, and one seroconverter had resistance to PIs.

The change in HIV diversity over time (HIV diversification) was evaluated for each participant by comparing HRM scores obtained at study enrollment to those obtained at the 12-month follow-up visit (end of study). End-of-study testing was performed for 70 (48%) of the 146 men who had HRM scores at enrollment (52 were lost to study follow-up and 24 had a viral load ≤400 copies/mL at the 12-month visit). HRM scores were obtained for all six regions for 62 (89%) of the 70 men (Fig 1). HIV diversification in each region was quantified as the difference between the HRM score at 12 months and the HRM score at study enrollment.

### Association of HIV diversity and duration of infection

**Fig 2:**
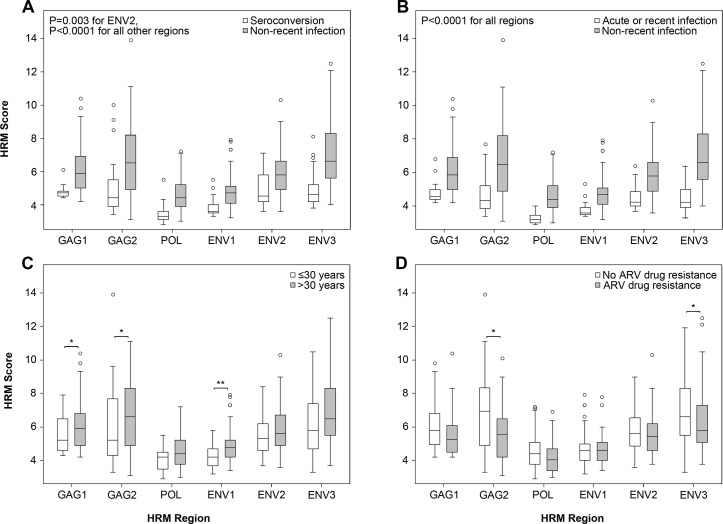
HRM scores for men who were HIV infected at enrollment and men who seroconverted during the HPTN 061 study. Box and whisker plots show the distribution of high resolution melting (HRM) scores for six regions in the HIV genome. Data are shown for men in different subgroups (acute infection at enrollment, recent infection at enrollment, non-recent infection at enrollment; seroconverters; see text). Symbols show the median (inner line), interquartile range (box), lower inner and upper outer fences (whiskers), and outliers (circles) for HRM scores. Univariate median regression analyses were used to compare HRM scores between the indicated groups; *p*-values for these comparisons are shown (*p<0.05; **p<0.01).

We compared HRM scores in three groups of men: (1) men who had acute or recent infection at enrollment (N = 16); (2) men who had non-recent infection at enrollment (N = 130); and (3) men who seroconverted during the study (N = 21, Table 1). In univariate analyses, median HRM scores were significantly lower in all six regions for seroconverters compared to men with non-recent infection (*p* = 0.003 for ENV2, *p*<0.0001 for all other regions, Fig 2A). Median HRM scores were also significantly lower in all six regions for men with acute or recent infection compared to men with non-recent infection (*p*<0.0001 for all regions, Fig 2B). In HPTN 061, younger men were more likely to acquire HIV infection [25] or be newly diagnosed [26]. In this study, younger age (≤30 years) was also associated with lower median HRM scores in GAG1 (*p* = 0.049), GAG2 (*p* = 0.031), and ENV1 (*p* = 0.002, Fig 2C). The results were still statistically significant after correcting for multiple comparisons.

We also analyzed the change in HIV diversity over time (HIV diversification) in the subset of 62 men who had HRM scores from both the enrollment and end-of-study visits. Overall, HRM scores increased over time in all six regions, with statistically significant increases in GAG1 (*p* = 0.03) and POL (*p*<0.0001). These results were still statistically significant after correcting for multiple comparisons.

HIV diversification in the POL region was significantly higher among men who had acute or recent infection at enrollment compared to those who had non-recent infection at enrollment (*p* = 0.029, Table 2). This association was no longer statistically significant after correcting for multiple comparisons.

**Table 2:** Association of HIV diversification with duration of HIV infection and other factors (paired analysis).

Characteristics at study enrollment	GAG1	GAG2	POL	ENV1	ENV2	ENV3
Acute or recent infection^a^	0.59	0.10	**0.029**	1.00	0.06	0.07
Age (years)	1.00	0.70	0.71	0.49	0.41	0.25
CD4 cell count (cells/mm^3^)	1.00	0.68	**0.033**	0.68	0.21	0.35
HIV viral load (log_10_)	0.28	0.44	**0.019**	0.75	0.32	1.00
ARV drug(s) detected (yes/no)	0.08	1.00	0.25	1.00	0.63	**0.049**
HIV drug resistance (yes/no)	0.70	1.00	0.65	1.00	1.00	0.76

HIV diversification was defined as the change in HRM scores over 12 months. Results were obtained for 62 men in HPTN 061 (see Fig 1). Median regression analysis was used to analyze the association of HIV diversification with various factors. The table shows *p*-values for these analyses; *p*-values <0.05 are bolded. Abbreviations: ARV: antiretroviral.

^a^ Acute or recent infection at study enrollment, compared to non-recent infection at study enrollment.

### Association of HIV diversity and other factors

**Fig 3:**
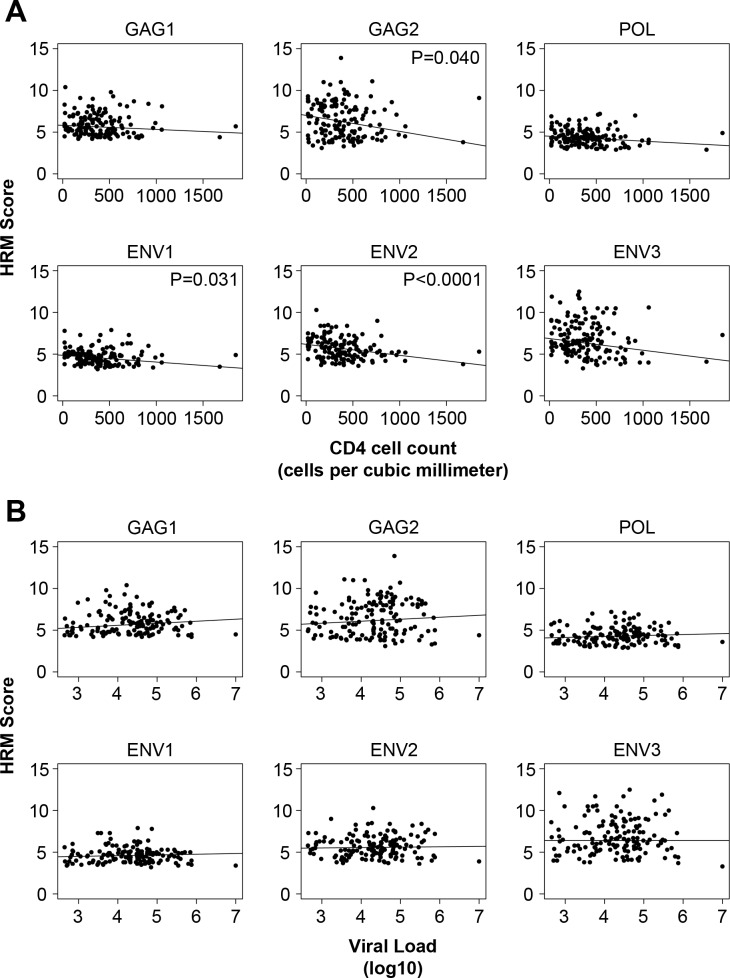
Association of HIV diversity with CD4 cell count and HIV viral load in men who were HIV infected at enrollment. Scatterplots show the relationship between high resolution melting (HRM) score with HIV viral load and CD4 cell count for men who were HIV infected at enrollment; *p*-values <0.05 are shown.

We next evaluated the association of HIV diversity at enrollment with other factors, including CD4 cell count, HIV viral load, ARV drug detection, and HIV drug resistance. In univariate analyses, higher CD4 cell count was associated with lower median HRM scores in GAG2 (*p* = 0.040), ENV1 (*p* = 0.031), and ENV2 (*p*<0.0001, Fig 3A). In contrast, HIV viral load was not significantly associated with HRM scores (Fig 3B). HIV drug resistance was associated with lower median HRM scores in GAG2 (*p* = 0.023) and ENV3 (*p* = 0.028, Fig 2D), and ARV drug detection was marginally associated with lower median HRM scores in POL (*p* = 0.047, data not shown). The same results were obtained after correcting for multiple comparisons.

HIV diversification in the POL region was significantly higher among men who had higher CD4 cell counts (*p* = 0.033) and lower HIV viral loads (*p* = 0.019) at study enrollment (Table 2). HIV diversification in the ENV3 region was lower among men who had ARV drugs detected at enrollment compared to men who did not have ARV drugs detected; however, this association was only marginally significant (*p* = 0.049). These associations were no longer statistically significant after correcting for multiple comparisons.

We explored associations between HIV diversity and other factors, including sexually transmitted infections and self-reported behaviors (prior or current ART use, substance use, multiple male partners, and unprotected receptive and insertive anal intercourse). None of these factors were significantly associated with HRM scores in any region (data not shown).

### Multivariate analysis of factors associated with HIV diversity

Multivariate analysis was performed for factors that were significantly associated with median HRM scores among men who were HIV infected at enrollment in the univariate analyses presented above. These factors included duration of HIV infection, age, CD4 cell count, ARV drug detection, and HIV drug resistance (Table 3). In this analysis, lower median HRM scores were associated with acute or recent infection (*p* = 0.0089 for GAG2, *p*<0.0001 for the other regions) and HIV drug resistance (*p* = 0.017 for GAG1 and *p* = 0.0003 for GAG2), after adjusting for the other factors. Weak associations were observed for lower median HRM scores and younger age (*p* = 0.046 for ENV1) and higher CD4 cell count (*p* = 0.049 for GAG2). HIV diversity was not associated with ARV drug detection in the multivariate analysis. Results from the multivariate analyses were still statistically significant after after correcting for multiple comparisons.

**Table 3:** Multivariate analysis of HIV diversity at study enrollment.

Covariate	Region	Estimate^a^	95% CI	P value
Acute or recent infection^b^	GAG1	-1.11	-1.51, -0.70	**<0.0001**
	GAG2	-1.46	-2.54, -0.37	**0.0089**
	POL	-1.08	-1.42, -0.73	**<0.0001**
	ENV1	-0.90	-1.18, -0.61	**<0.0001**
	ENV2	-1.25	-1.85, -0.66	**<0.0001**
	ENV3	-1.90	-2.76, -1.05	**<0.0001**
Age ≤30 years	GAG1	0.05	-0.43, 0.52	0.85
	GAG2	-1.06	-2.13, 0.01	0.05
	POL	-0.23	-0.55, 0.08	0.15
	ENV1	-0.29	-0.57, -0.005	**0.046**
	ENV2	-0.07	-0.64, 0.50	0.81
	ENV3	-0.39	-1.24, 0.46	0.37
CD4 cell count	GAG1	-0.0002	-0.0012, 0.0008	0.71
	GAG2	-0.0009	-0.0018, 0	**0.049**
	POL	-0.0002	-0.0007, 0.0003	0.45
	ENV1	-0.0001	-0.0007, 0.0005	0.66
	ENV2	-0.0007	-0.0014, 0.0001	0.10
	ENV3	-0.0004	-0.0017, 0.0008	0.49
ARV drug(s) detected	GAG1	-0.14	-0.56, 0.28	0.50
	GAG2	-0.13	-0.96, 0.70	0.76
	POL	-0.04	-0.36, 0.28	0.80
	ENV1	0.09	-0.19, 0.36	0.53
	ENV2	0.19	-0.32, 0.70	0.47
	ENV3	-0.10	-0.85, 0.65	0.80
HIV drug resistance	GAG1	-0.54	-0.99, -0.10	**0.017**
	GAG2	-1.69	-2.57, -0.80	**0.0003**
	POL	-0.19	-0.55, 0.17	0.29
	ENV1	-0.08	-0.38, 0.23	0.61
	ENV2	-0.31	-0.87, 0.26	0.28
	ENV3	-0.70	-1.49, 0.10	0.09

Multivariate analysis was performed separately for each HRM region using the covariates listed; *p*-values <0.05 are bolded. Abbreviations: CI: confidence interval; ARV: antiretroviral.

^a^ The estimate shows the difference in median HRM score for each factor after adjusting for covariates.

^b^ Acute or recent infection at study enrollment, compared to non-recent infection at study enrollment.

## Discussion

In previous studies, we used the HRM diversity assay to analyze HIV diversity in pediatric and adult cohorts. This report extends those studies by examining HIV diversity among HIV-infected Black MSM who had high levels of non-suppressive ARV drug use and HIV drug resistance [28]. In this study, lower viral diversity was strongly associated with a shorter duration of HIV infection; lower HRM scores were observed in men who had acute or recent infection at enrollment, men who seroconverted during the study, and men who were younger at study enrollment. Notably, the association of lower HIV diversity with acute or recent infection was independent of ARV drug use and HIV drug resistance. The patterns of HIV diversity in *env*, *gag*, and *pol* observed among men with documented HIV seroconversion (tested six months after their last HIV negative test) were nearly identical to those classified as recently infected at enrollment using a MAA developed for HIV incidence estimation. This is consistent with our previous study where individuals with known recent infection had lower HRM scores than those with known non-recent infection [17]. The findings in this report provide further data supporting the use of the MAA to identify individuals with recent HIV infection for research studies [18].

We also analyzed the change in HIV diversity over time (HIV diversification). HIV diversity increased over time in all six regions for men who had acute or recent infection at study enrollment. However, when those results were compared to results from men who had non-recent infection at enrollment, a significant difference in diversification was only observed for the HIV *pol* region. In a different US cohort, HIV *pol* diversified 30-fold more among individuals with recent vs. chronic HIV infection when a sequence-based diversity measure was used [8]. We found somewhat different results in a cohort of women from Malawi. In that study, higher levels of HIV diversification were observed in women with recent vs. non-recent HIV infection for *gag* and *env*, but not *pol* [18]. A novel finding in this report was the association between HIV diversification with lower CD4 cell count and higher HIV viral load.

We also analyzed the association of HIV viral load and viral diversity. While it is possible sampling error in low viral load samples could affect HIV diversity measures, we previously demonstrated that HRM scores are not affected by viral load, provided that >100 copies of HIV RNA is used for analysis [21]. In this report, 203 (89%) of the 229 samples analyzed had >100 copies of input HIV RNA. There was no association between HIV diversity and viral load among men who were HIV infected at study enrollment, and lower HIV *gag* diversity was only weakly associated with higher CD4 cell count after adjusting for other factors. These findings are consistent with other studies that reported no association of HIV diversity with viral load [13,20,21] or CD4 cell count [11,17]. We did find that HIV *pol* diversified more over 12 months among men who had higher CD4 cell counts and lower HIV viral loads at study enrollment. These factors may be surrogates for a shorter duration of HIV infection. Additional studies are needed to understand factors associated with diversification in different regions of the HIV genome.

We did not find associations between HIV diversity or diversification and ARV drug use. It is possible that the men who had ARV drugs detected did not have prolonged exposure to non-suppressive ARV drug levels; of note, approximately half of the men who had ARV drugs detected at study enrollment did not have HIV drug resistance [28]. A limitation of this study is that ARV drug testing was only performed for enrollment samples; some men may have taken ARV drugs during study follow-up, which could have impacted HIV diversification. Lower diversity in both HIV *gag* regions analyzed was independently associated with resistance to NRTIs, NNRTIs, and/or PIs. This finding was surprising, since these ARV drug classes target enzymes encoded by HIV *pol* (protease and reverse transcriptase). Prolonged exposure to non-suppressive ART has been significantly associated with decreases in HIV diversity in *pol* in adults [32] and in *gag*, *pol*, and *env* in children [19]. Lower *gag* diversity was also associated with self-reported prior maternal ARV drug use in a cohort of African children [20]. These findings suggest that the selective pressure exerted by ARV drugs may influence HIV diversity in regions other than *pol*. The 225-base pair *pol* region analyzed in this study spans the junction between the HIV protease and HIV reverse transcriptase coding regions [17] and has the lowest level of genetic diversity of the six regions analyzed. This region contains many PI-associated resistance mutations but does not contain NRTI- or NNRTI-associated resistance mutations.

Most studies of HIV diversity have analyzed *env* [3,9,10,33] or *gag* and *env* [4,6,7]. The high-throughput HRM diversity assay used in this study can be used to analyze multiple genomic regions, providing a more complete picture of HIV diversity and diversification over time. In this study, we analyzed HIV diversity in six regions of the HIV genome for 167 men, including two time points for 62 men (>1300 measures overall). Some of the associations we observed for HIV diversity and diversification were not detected in studies with more limited analyses. Further studies of HIV diversity and diversification may yield new insights into the selective pressures driving viral evolution in different populations and settings.

## Supporting Information

S1 File High resolution melting (HRM) scores and demographic, behavioral, and clinical factors of HIV-infected men analyzed using the HRM diversity assay in HPTN 061. (CSV)
